# Tissue-specific reductions in mitochondrial efficiency and increased ROS release rates during ageing in zebra finches, *Taeniopygia guttata*

**DOI:** 10.1007/s11357-022-00624-1

**Published:** 2022-08-19

**Authors:** Pablo Salmón, Caroline Millet, Colin Selman, Pat Monaghan, Neal J. Dawson

**Affiliations:** School of Biodiversity, One Health and Veterinary Medicine, University of Glasgow, Graham Kerr Building, Glasgow, G12 8QQ UK

**Keywords:** Proton leak, Senescence, Birds, Mitochondrial physiology, Reactive oxygen species, Ageing

## Abstract

Mitochondrial dysfunction and oxidative damage have long been suggested as critically important mechanisms underlying the ageing process in animals. However, conflicting data exist on whether this involves increased production of mitochondrial reactive oxygen species (ROS) during ageing. We employed high‐resolution respirometry and fluorometry on flight muscle (pectoralis major) and liver mitochondria to simultaneously examine mitochondrial function and ROS (H_2_O_2_) release rates in young (3 months) and old (4 years) zebra finches (*Taeniopygia guttata*). Respiratory capacities for oxidative phosphorylation did not differ between the two age groups in either tissue. Respiratory control ratios (RCR) of liver mitochondria also did not differ between the age classes. However, RCR in muscle mitochondria was 55% lower in old relative to young birds, suggesting that muscle mitochondria in older individuals are less efficient. Interestingly, this observed reduction in muscle RCR was driven almost entirely by higher mitochondrial LEAK-state respiration. Maximum mitochondrial ROS release rates were found to be greater in both flight muscle (1.3-fold) and the liver (1.9-fold) of old birds. However, while maximum ROS (H_2_O_2_) release rates from mitochondria increased with age across both liver and muscle tissues, the liver demonstrated a proportionally greater age-related increase in ROS release than muscle. This difference in age-related increases in ROS release rates between muscle and liver tissues may be due to increased mitochondrial leakiness in the muscle, but not the liver, of older birds. This suggests that age-related changes in cellular function seem to occur in a tissue-specific manner in zebra finches, with flight muscle exhibiting signs of minimising age-related increase in ROS release, potentially to reduce damage to this crucial tissue in older individuals.

**Supplementary Information:**

The online version contains supplementary material available at 10.1007/s11357-022-00624-1.

## Introduction

Understanding the mechanisms responsible for age-related declines in physiological performance and concomitant increases in mortality risk is an important area of research across many scientific disciplines [1, 2]. One of the long-standing mechanistic hypotheses of ageing is that tissue deterioration occurs as a consequence of progressive accumulation of unrepaired damage to biomolecules inflicted by reactive oxygen species (ROS) [3]. While organisms have evolved antioxidant defences and tissue repair capabilities that provide resistance to age-induced increases in oxidative damage (e.g. reviewed in [4, 5]), there remains ambiguity as to the ubiquity of age-related oxidative damage (e.g. [6–13]).

One area of research that has gained substantial attention is centred on the mitochondrial production of ROS. Mitochondria account for the vast majority of ROS production within animal cells ([14], but see [15]). Mitochondrially generated ROS results from the leakage of electrons from the electron transport system (ETS), producing short lived, but highly reactive, free radicals such as superoxide (O_2_^•–^) [16]. Superoxide, in turn, is converted to hydrogen peroxide (H_2_O_2_) by superoxide dismutase (SOD) [17, 18]. Hydrogen peroxide is a relatively stable, membrane permeable molecule capable of diffusing out of the mitochondrial matrix [19]. The majority of recent studies exploring mitochondrial ROS production have centred on the rates of hydrogen peroxide release from the mitochondria, with many comparative studies linking variation in ROS release and oxidative damage with variation in lifespan [20–23]. Furthermore, ROS production rates can vary greatly between different tissues [24], which may suggest that any age-related increases in ROS production may affect certain tissues more than others [25].

This study examined the physiology of mitochondria from the flight muscle and liver of young (3 months) and old (4 years) zebra finches. Our study species, the zebra finch (*Taeniopygia guttata*), is a songbird commonly used in laboratory studies; in captivity, it displays both an age-associated decline in reproductive success [26, 27] and an age-associated increase in mortality rate from around 3 years of age [27]. We examined mitochondrial respiration and reactive oxygen species (ROS) emission using a substrate titration protocol to assess relative contribution of individual complexes of the electron transport system (complex I or II substrates), and integrated mitochondrial function with a fully reconstituted tricarboxylic acid cycle (complex I and II). We set out to determine if mitochondrial physiology deteriorates in older adult zebra finches, and, by studying both the flight muscle and liver, we aimed to uncover whether these age-related changes are conserved across tissues or are effects that are tissue-specific. We hypothesised that mitochondrial physiology deteriorates in older finches, predicting that older finches will exhibit lower mitochondrial respiratory efficiency and greater ROS release rates relative to younger birds.

## Methods

### Animals and mitochondrial homogenate preparations

We used zebra finches from our captive, out-bred stock population at the University of Glasgow. All birds were reared and subsequently maintained under standardised conditions in captivity. Young zebra finches were around 3 months old (median: 82 days; range: 10–12 weeks; *n* = 12) while the old zebra finches were approximately 4 years old (median: 1312 days; range: 3.6–4.3 years; *n* = 12). The maximum lifespan in the wild for zebra finches is 5 years, with few birds living beyond 7 years even in captivity [28–31]. Birds were maintained under constant ambient temperature (20–24 °C) and photoperiod (14L:10D) and with *ad libitum *access to food and water. All housing conditions and protocols were approved by and carried out under UK Home Office Project Licence (70/8335) and following local ethical review.

Individual zebra finches were humanely euthanized by cervical dislocation following UK Home Office guidelines and subsamples of the flight muscle (pectoralis major) and liver were immediately collected, weighed, and homogenised in ice cold MiR05 respirometry buffer (0.5 mM EGTA, 3 mM MgCl_2_, 60 mM K-lactobionate, 20 mM taurine, 10 mM KH_2_PO_4_, 20 mM Hepes, 110 mM sucrose, free fatty acid bovine serum albumin (1 g L^−1^), pH 7.1) for analyses of the mitochondrial properties. Total liver mass was measured at the time of tissue collection, but it was not possible to measure pectoral muscle mass for logistical reasons. However, we were able to measure pectoral muscle mass on a separate sample of zebra finches of the same age classes (young: median = 78.4 days, range = 75–83 days, *n* = 10; old: median = 1392 days, range = 1373–1463 days, *n* = 16). We used scaled mass index, a proxy for body condition, calculated using tarsus length according to Peig and Green [32]:

$$\mathrm{Scaled\; mass\; index}\hspace{0.17em}=\hspace{0.17em}{M}_{i} {[{L}_{o}/{L}_{i}]}^{b\mathrm{_{SMA}}}$$

where *M*_*i*_ is the body mass; *L*_*i*_ is the length of the tarsus; *L*_*o*_ is the mean length of tarsi for all birds used in this study; and the scaling exponent *b*_SMA_ is calculated by dividing the slope of the ordinary least squares regression by Pearson’s correlation coefficient [32]. The muscle and liver preparations were undertaken using a modified version of Salin et al [33]. Briefly, tissues were minced using micro-dissection scissors to obtain a homogeneous solution in 2 mL of MiR05 and subsequently mixed using a Dounce homogeniser at 100 rpm (Cole-Parmer PTFE Tissue Grinder, Cambridgeshire, UK) for 2 up-down cycles (staying for 30 s on ice in between). The entire procedure was carried out on ice and completed within 1 h of the bird being culled.

### Mitochondrial respiration and ROS release rates

Rates of oxygen consumption and H_2_O_2_ production were monitored simultaneously using an Oxygraph-2k high-resolution respirometer (Oroboros, Innsbruck, Austria) equipped with a fluorescence detection module mounted with excitation and release rate filters for the fluorescent probe Amplex Ultra Red (AUR). A total of 500 μg of tissue (muscle or liver) was added to 2 mL of respiratory buffer (MiR05) previously air-equilibrated at 41 °C in the Oroboros chamber and under continuous stirring. AUR (10 μM), horseradish peroxidase (5 IU/mL), and superoxide dismutase (25 UI/mL) were added to the chamber at the beginning of each sequence. The mitochondrial respiration assay was started by adding malate (10 mM) followed by pyruvate (5 mM) to stimulate LEAK-state respiration (LEAK). ADP (5 mM) was added to elicit oxidative phosphorylation respiration (OXPHOS) via complex I (*P*_PM_). In order to obtain the maximal capacity for OXPHOS via complexes I + II, glutamate (24 mM; *P*_PMG_) and succinate (10 mM; *P*_PMGS_) were sequentially added. Cytochrome *c* (10 μM) was then added to assess the viability of the preparations (outer-mitochondrial-membrane integrity). The addition of exogenous cytochrome *c* resulting in a less than 10% increase in mitochondrial respiration was considered acceptable [34], confirming the quality of the mitochondrial preparation for each tissue (muscle: 5.1 ± 8.4%; liver: 0.5 ± 3.9%; mean ± SD; muscle: *t*_65.5_ =  − 0.16, *p* = 0.873, liver: *t*_65.5_ = 0.03, *p* = 0.976). Antimycin A (5 mM) was then added to account for residual or non-mitochondrial oxygen respiration. Finally, the maximal activity of complex IV was measured after the addition of ascorbate (2 mM) and *N*,*N*,*N*ʹ,*N*ʹ-tetramethyl-p-phenylenediamine dihydrochloride (TMPD, 0.5 mM; *P*_Tm_). Rates of fluorescent product (resorufin) formation were converted to nmoles of H_2_O_2_ based on a two‐point standard curve conducted at the beginning and end of each assay by direct addition of H_2_O_2_ in the chamber, thereby accounting for chemical interference and fluorescence quenching by mitochondria. The respiratory control ratio (RCR) was calculated by calculating the ratio of maximal OXPHOS respiration rate (*P*_PMGS_) relative to LEAK-state respiration (LEAK).

### Citrate synthase assays

Citrate synthase activity (CS), a commonly used marker of mitochondrial volume [35], was assayed at 41 °C following Dawson et al. [36]. Muscle or liver tissue was kept on ice and homogenised in homogenising buffer (100 mmol L^−1^ KH_2_PO_4_ buffer, pH 7.2, containing 1 mmol L^−1^ EGTA, 1 mmol L^−1^ EDTA, and 1 mmol L^−1^ phenylmethylsulfonylfluoride (PMSF)). The homogenates were then centrifuged at 1000 g at 4 °C and the resultant supernatant was used. Assays were conducted in triplicate (intra-assay technical repeatability based on Lessells and Boag [37]: 0.98, 95% CI [0.96–0.99], *p* < 0.001) at 412 nm (*ε* = 14.15 (mmol L^−1^)^−1^) in 100 mmol L^−1^ KH_2_PO_4_ (pH 7.2), 0.15 mmol L^−1^ acetyl-CoA, 0.15 mmol L^−1^ 5,5′-dithiobis-2-nitrobenzoic acid, and 0.5 mmol L^−1^ oxaloacetate (omitted in blank). All assays were run on a SpectraMaxPlus 384 spectrophotometer (Molecular Devices, San Jose, CA, USA), and data were analysed using the accompanying SoftMax Pro 6.3 program.

### Statistical analysis

All analyses were performed in R 3.5.2. [38]. We fitted independent linear mixed models for each tissue (muscle or liver) in order to compare the role of age category (3 months and 4 years) in the oxygen consumption (per mg of tissue and normalised by CS) and H_2_O_2_ release rates (including ROS release/O_2_ consumption expressed as a percentage of respirational O_2_ flux in each respiration state). All models included age category, respiration state, sex, and the interaction “*age category* × *respiration state*” as fixed effects and individual identity as a random effect. Sexes were balanced in our sample (young: 6 males and 6 females; old: 7 males and 5 females); thus, we did not include the interaction “*sex* × *age category*” in order to avoid overparameterisation. When the interaction “*age category* × *respiration state*” was found significant, post hoc tests with Tukey–Kramer HSD correction were used to provide *P* values for the pair-wise comparisons. A similar model structure was used to assess the effects of age on the respiratory control ratio (RCR); in this case, the model included age category, tissue, sex, and the interaction “*age category* × *tissue*” as fixed effects and individual identity as a random effect. The effects of body mass, scaled body mass index, liver mass, and estimated pectoral mass were analysed using linear models with sex and age category as fixed effects. Homoscedasticity and normality were ascertained by visual examination of the residual distribution. Dependent variables were transformed when necessary (see Supplementary Tables S1–S3 for further details). Overall model explanatory power in mixed models was calculated as marginal (*R*^2^_*m*_) and conditional (*R*^2^_*c*_) values [39] and the significance of parameter estimates was estimated using conditional *F*‐tests based on Satterthwaite approximation for the denominator degrees of freedom. In all cases, *P* < 0.05 was considered significant; sample size is presented in figure legends and supplementary tables. The results in the figures are presented as individual raw data points together with the mean and standard error.

## Results

### Body mass, body condition, liver mass, and pectoral mass

At sampling, young (3 months) and old (4 years) individuals did not differ in either body mass (*F*_1,21_ = 0.79, *P* = 0.384; Supplementary Table S1, Figure S1a) or scaled mass index (*F*_1,21_ = 1.16, *P* = 0.293; Supplementary Table S1, Figure S1b). In addition, total liver mass was similar between both groups (*F*_1,21_ = 0.11, *P* = 0.918; Supplementary Table S1, Figure S1c). Due to sampling limitations, we were unable to obtain pectoral masses from those individuals used to measure mitochondrial function. However, we measured pectoral masses from a separate sample of zebra finches of the same age classes (young: median = 78.4 days, range = 75–83 days, *n* = 10; old: median = 1392 days, range = 1373–1463 days, *n* = 16) which showed no differences between pectoral mass for old versus young birds (*F*_1,24_ = 1.04, *P* = 0.317; Supplementary Table S1, Figure S1d). No sex differences were observed in any morphometric trait (all *P* > 0.683).

### Mitochondrial physiology

**Fig. 1:**
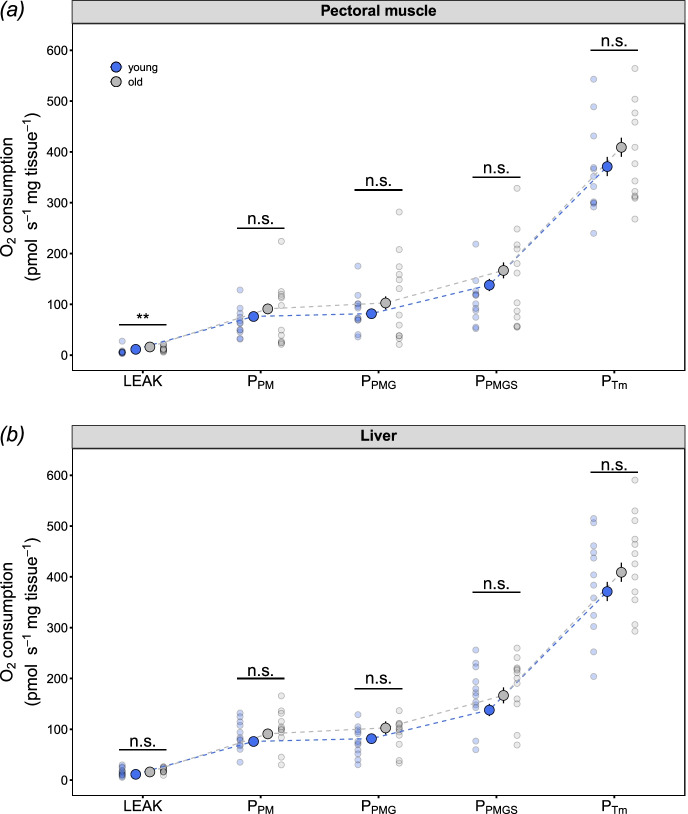
Mitochondrial O_2_ consumption rates from young and old zebra finches in **a** pectoral muscle and **b** liver tissues. LEAK = pyruvate + malate; *P*_PM_ = pyruvate + malate + ADP; *P*_PMG_ = pyruvate + malate + glutamate + ADP; *P*_PMGS_ = pyruvate + malate + glutamate + succinate + ADP; *P*_Tm_ = TMPD + ascorbate. Blue circles = young finches; grey circles = old finches. Significant differences between age categories were assessed by the Tukey-HSD test or main effects (see text and Supplementary Table S2 for details), with **P < 0.01. Data are raw data points and means ± SE, *n* = 12 for both young and old finches

**Fig. 2:**
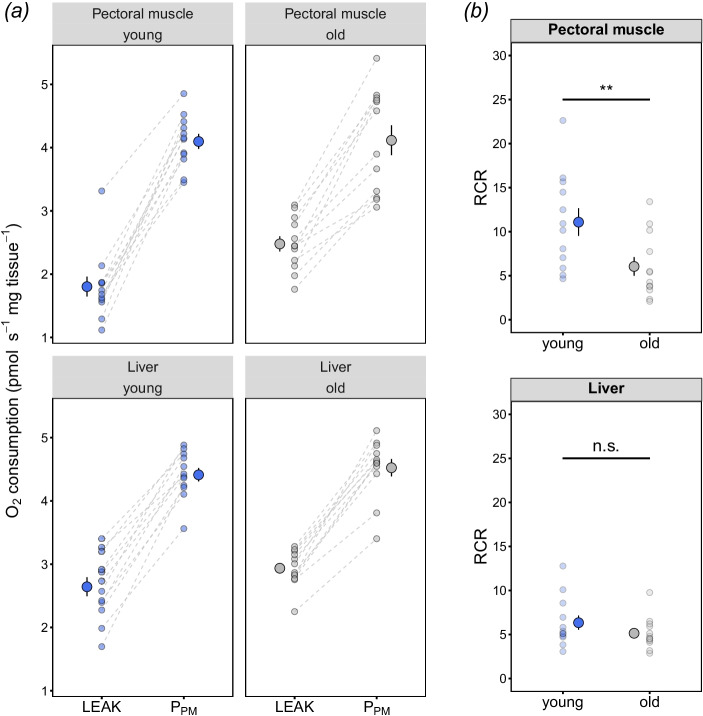
Mitochondrial respiratory control ratios (RCR) from young and old zebra finches in pectoral muscle and liver. Within-individual change in **a** O_2_ consumption between LEAK and *P*_PM_ states per age category in pectoral muscle and liver; **b** RCR for pectoral muscle and liver. In **a**, *y*-axis is log-transformed to facilitate the visualisation and interpretation of the data (see text for details) and dashed lines connect points of the same individual while the bigger points represent the mean per respiration state. LEAK = pyruvate + malate; *P*_PM_ = pyruvate + malate + ADP; RCR = *P*_PM_/LEAK. Blue circles = young finches; grey circles = old finches. Significant differences between age categories were assessed by the Tukey-HSD test or main effects (see text for details), with **P < 0.01. Data are raw data points and means ± SE, *n* = 12 for both young and old finches

Mitochondrial oxygen consumption in young and old zebra finches differed between respiratory states in skeletal muscle (*age category* × *respiration state*: *F*_4,88_ = 4.51, *P* = 0.002; Fig. 1a) but not in liver (*age category* × *respiration state*: *F*_4,88_ = 0.735, *P* = 0.571; Supplementary Table S2; Fig. 1b). Post hoc analyses (Tukey’s HSD) indicate that the muscle in old individuals had higher oxygen consumption rates during LEAK-state respiration (*t*_37_ = 3.02, *P* = 0.005; Fig. 2a). However, no further differences were observed after the addition of ADP to initiate OXPHOS respiration (*P*_PM_), nor during the subsequent stimulation with glutamate (*P*_PMG_) and succinate to obtain the maximal capacity for OXPHOS via complexes I + II (*P*_PMGS_; *P* > 0.380; Fig. 1a). Maximal oxygen consumption rate via complex IV (*P*_Tm_) did not differ between ages in either muscle or liver (*P* > 0.485). Mitochondrial respiration rates were also normalised to mitochondrial volume (CS activity), and showed a similar pattern to uncorrected respiration rates, where only LEAK-state respiration was elevated in old finches when compared to young finches (Supplementary Table S2 and Figure S2). However, the respiratory control ratio (RCR; the ratio of maximal OXPHOS respiration rate relative to LEAK-state respiration) significantly differed between age categories (*age category* × *tissue*: *F*_1,21_ = 14.74, *P* < 0.001), with RCR in older birds being lower than young birds in muscle (*t*_29_ = 4.72, *P* = 0.001; Fig. 2b) but not different in the liver (*t*_29_ = 1.51, *P* = 0.142; Fig. 2b). Further analyses indicate that the differences in muscle RCR were due to a greater LEAK respiration in older birds, rather than any decrease in maximal OXPHOS respiration (*P*_PMGS_).

### ROS release rates

**Fig. 3:**
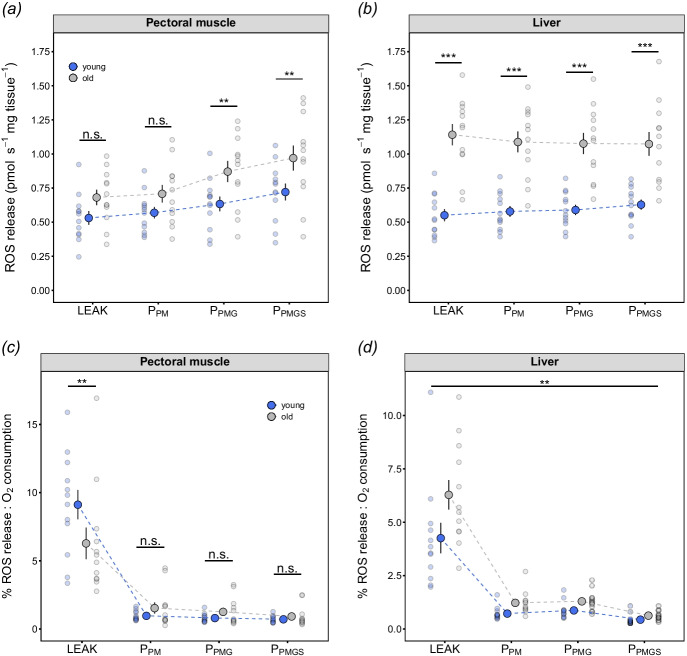
Mitochondrial ROS release rates (H_2_O_2_) from young and old zebra finches in **a** pectoral muscle and **b** liver tissues; and ROS release rate expressed as a percentage of O_2_ consumption in **c** pectoral muscle and **d** liver tissues. LEAK = pyruvate + malate; *P*_PM_ = pyruvate + malate + ADP; *P*_PMG_ = pyruvate + malate + glutamate; *P*_PMGS_ = pyruvate + malate + glutamate + succinate; *P*_Tm_ = TMPD + ascorbate. Blue = young; grey = old. Significant differences between age categories were assessed by the Tukey-HSD test or main effects (see text and Supplementary Table S3 for details), with **P < 0.01 and ***P < 0.001. Data are raw data points and means ± SE, *n* = 12 for both young and old finches

In muscle, we found a significant interaction between age and respiration state (*F*_3, 66_ = 3.28, *P* = 0.026; Supplementary Table S2). Subsequent post hoc tests indicated that the differences in ROS release rates between young and old individuals were not homogeneous across respiratory states. Indeed, muscle mitochondria H_2_O_2_ release rates were similar across young and old birds during LEAK-state respiration and initial OXPHOS respiration rates using pyruvate and malate (*P* > 0.115; Fig. 3a). However, the subsequent addition of glutamate (*P*_PMG_) and succinate (*P*_PMGS_) led to a significant increase in H_2_O_2_ release rates in the old birds (*P*_PMG_: *t*_25.1_ = 2.57, *P* = 0.016; *P*_PMGS_: *t*_25.1_ = 2.69, *P* = 0.012; Fig. 3a). In contrast, H_2_O_2_ release rates in liver mitochondria were significantly higher in the old birds across all respiratory states (all *P* < 0.001; Fig. 3b).

The simultaneous measurement of H_2_O_2_ release rate and of O_2_ consumption allowed us to calculate ROS release/O_2_ consumption in each of the respiration states. There was again a significant interaction between age category and respiration state in ROS release/O_2_ consumption for LEAK-state respiration within muscle mitochondria (Supplementary Table S3). Post hoc tests showed that the differences between young and old individuals were only significant during LEAK-state respiration, when younger individuals had a higher ROS release/O_2_ consumed in the LEAK state (LEAK: *t*_75.2_ = 3.32, *P* = 0.001; *P*_PM_: *t*_75.2_ =  − 0.69, *P* = 0.491; *P*_PMG_: *t*_75.2_ =  − 0.56, *P* = 0.579; *P*_PMGS_: *t*_75.2_ =  − 0.27, *P* = 0.789; Fig. 3c). In contrast, the ROS release/O_2_ consumption within liver mitochondria was significantly higher in the old birds across all mitochondrial respiratory states (*F*_1, 21_ = 9.38, *P* = 0.006; Supplementary Table S2; Fig. 3d).

## Discussion

Mitochondrial dysfunction is one of the hallmarks of ageing [40] and is manifested by a combination of impaired oxidative phosphorylation (OXPHOS) activity, increased oxidative damage, decline in mitochondrial quality control, and reduced activity of antioxidant enzymes, coupled to changes in mitochondrial morphology, dynamics, and biogenesis [41–43]. Our present findings provide evidence of mitochondrial dysfunction in zebra finches during ageing but that the effects appear tissue-specific. Mitochondria from flight muscle, but not the liver, showed a reduced respiratory control ratio (RCR; a measure of the coupling state of mitochondria) during ageing. This change in the coupling state of muscle mitochondria was largely driven by increases in the LEAK-state respiration. In addition, mitochondria from both the muscle and liver of old zebra finches showed increased ROS release rates when compared to the younger birds. Taken together, our results suggest that increased ROS release may be a feature of mitochondrial dysfunction in older finches; yet, muscle tissue demonstrated a comparatively smaller increase in age-related ROS release. This smaller increase in ROS release rates in muscle mitochondria may be driven by increased mitochondrial leakiness, where age-related increases in ROS release rates may be mitigated by a reduction in coupling efficiency.

### Mitochondrial coupling efficiency is lower in muscle mitochondria from old zebra finches

Zebra finch muscle mitochondria showed a decrease in efficiency as evidenced by a reduction in RCR in old individuals when compared to young individuals. RCR has been suggested as one of the most important indicators of mitochondrial function as any changes in either oxidative phosphorylation or LEAK-state respiration can change RCR [44]. Furthermore, sarcopenia—the atrophy of skeletal muscle and, consequently, a decline in muscle strength—is regarded as a key consequence of the ageing process [45]. While the proximate mechanisms underlying sarcopenia are still largely unclear, dysfunctional muscle mitochondrial activity has been suggested to initiate the signalling cascade leading to age-related motor neuron and muscle fibre death (e.g. [46]). The current literature regarding age-related changes in muscle performance or its underlying mechanisms in avian systems is scarce (e.g. [47–50]), but we expect that birds will prioritise the maintenance of flight muscle to sustain the ability to fly, and ultimately improve survival. In captive Japanese quail (*Coturnix japonica*), single wing loading (relative to contralateral unloaded wing) induced greater pectoralis muscle hypertrophy in young compared to old birds and higher H_2_O_2_ production in muscle in the older individuals after 7 days, but returned to baseline levels after 21 days [51]. This may suggest that there is an impairment of cellular ROS handling in the muscle of older quails, either due to increased ROS production and/or an attenuated ability to detoxify cellular ROS. Therefore, the tissue accumulation of ROS may be a major contributor to a decline in muscle performance in older birds. However, it is also possible that differential survival of specific phenotypes contributed to this effect.

### Release of reactive oxygen species is higher from muscle and liver mitochondria of old zebra finches

Across both muscle and liver tissues, the old zebra finches showed elevated mitochondrial ROS release rates when compared to the young birds. It has been suggested that an age-related decline in mitochondrial function, triggered by increasing mitochondrial ROS production, might affect fitness and longevity (e.g. [5, 52]). In a study exploring oxidative damage to flight muscle due to sustained migratory flight in Yellow-rumped warblers (*Setophaga coronata*), the authors found that simulated migratory flight resulted in an increase in oxidative damage to proteins (increased protein carbonyls) [53]. Additionally, oxidative damage to DNA and proteins in blood increases with chronological age from mid/early adulthood in zebra finches [54]. However, although age-associated increases in tissue oxidative damage have been repeatedly suggested as being associated with increases in mitochondrial ROS production [55–57], there is still little direct evidence that ageing increases mitochondrial ROS release rates in non-model organisms (e.g. [58]). In fact, there is evidence that mitochondria could be net consumers of oxygen radicals [59], and flight capable species of birds, which have high mitochondrial density in flight muscle, may have an increased capacity to scavenge ROS when compared to non-flying bird species [60]. However, our ROS measurements reflected the release of hydrogen peroxide from mitochondria, ultimately representing the ROS that escapes mitochondrial antioxidant mechanisms and leaks from the mitochondria [61]. In our birds, we cannot rule out that reductions in antioxidant activity due to age, in particular a reduction in the activity of mitochondrial peroxidases (e.g. catalase, glutathione peroxidase), may be influencing higher ROS release rates in older individuals; yet, early studies in laboratory rodents suggest that certain antioxidant activities (mitochondrial superoxide dismutase and catalase) increase with age in a tissue-specific manner and this seems to occur despite higher age-related ROS release rates [62–64]. It may also be possible that observed age differences are due to differential survival of particular phenotypes into old age since ours is a cross-sectional study. However, our results show no significant change in the variation of ROS release rates observed between age groups, suggesting that particular ROS release phenotypes have not been lost. The few studies that have explored age-related antioxidant changes in birds typically show no change (e.g. [48, 65]), with a study in zebra finches actually showing that superoxide dismutase levels in plasma increases with age [54]. While this may or may not be indicative of changes in superoxide dismutase levels other tissues in the zebra finches, taken together with the fact that ROS levels increase in both tissues measured here, it strengthens the idea that the observed increase in ROS release may be due to increased mitochondrial ROS production and not due to a decline in antioxidant defence capacity. In muscle tissue, increased ROS production during exercise can lead to an increase in antioxidants (reviewed in [66]); however, this is a transient increase in ROS levels and our result suggests a constitutively higher level of mitochondrial ROS release rates from older individuals. Therefore, the increased levels of antioxidant enzymes observed during ageing by Marasco et al. may be a compensatory mechanism to counter age-related increases in mitochondrial ROS release rates in older birds [54]. Regardless of the state of antioxidant defences, the higher ROS release rates observed in both the muscle and liver of older individuals may suggest that increased ROS release is evidence of mitochondrial dysfunction during ageing in finches.

### Tissue-specific differences in age-related mitochondrial dysfunction

It is possible that the lower RCR observed in the muscle but not the liver represents a sacrifice in muscle mitochondrial functionality to mitigate ROS release, and thereby tissue damage, through a reduction in coupling efficiency. Although we can only speculate regarding the link between mitochondrial efficiency and flight performance, it is expected that the maintenance of an efficient musculoskeletal flight system is a critical aspect to ensure survival in birds, as in the wild, the costs of a suboptimal performance, e.g. low foraging efficiency or poor predatory escape-behaviour, would be strongly selected against. This is not the first suggestion of compensatory mechanisms in zebra finch mitochondria during ageing. We have previously shown that senescent birds, over 6 years old, seem to increase mitochondrial quantity in red blood cells potentially to mitigate a decline in mitochondrial quality [66]. Age-related changes in muscle mitochondrial content remain a controversial topic, with biomedical oriented studies showing reduced (e.g. [68, 69]) or no changes in mitochondrial content with age (e.g. [70, 71]). Our data does not suggest a decline in muscle mass in older individuals, inferred by the lack of change in body mass and body condition between young and old finches, and due to no significant difference in pectoral muscle mass observed in a separate sampling of young and old finches (Figure S1d), which suggests that muscle loss associated with sarcopenia is unlikely to be occurring in our older finches. It is possible that other mechanisms previously described for other taxa, such as changes in the mitochondrial ultrastructure [72] or changes in the muscle fibre type and/or size [70], might provide alternative compensatory mechanisms. However, the current information regarding age-related muscle composition in birds is limited to studies in long-lived groups such as seabirds, finding no differences in muscle fibre size [47, 48], although there was an observed decline in the myonuclear domain by Jiménez et al. [48] which may represent declining muscle fibre performance due to ageing. It may also be possible that zebra finch muscle tissue accumulates ROS at a higher rate than other tissues like RBCs or the liver due to slower cellular turnover rates [73]. This would further increase the importance of a compensatory mechanism specific to muscle tissue in order to reduced long-term build-up of ROS damage. We did not observe similar changes in mitochondrial quality in the liver as we observed in flight muscle, or previously observed in red blood cells [67]. This is not entirely unexpected as age-related changes in liver function in mammals are most often attributed firstly to a reduction in liver size (not seen in our study; Figure S1c) due to inadequate tissue regeneration before the onset of any decline in mitochondrial function (e.g. [74–77]).

## Conclusion

Our study shows for the first time in an avian model tissue-specific differences between young and old birds in mitochondrial functionality. This is attributable to an increase in ROS release rates shared across muscle and liver tissues, although flight muscle mitochondria also show signs of reduced mitochondrial efficiency as evidenced by a reduced RCR and greater LEAK-state respiration in older individuals. Flight muscle mitochondria show evidence of reduced efficiency in older individuals, which could potentially have implications for sustaining flight which might be traded off against muscle damage. As mentioned above, our results are based on cross-sectional comparisons and further studies exploring this phenomenon throughout an individual’s lifetime, as well as measuring flight performance or muscle mechanics, are necessary.

## Supplementary Information

Below is the link to the electronic supplementary material.

Supplementary file1 (DOCX 424 KB)
