# Supplementary material for: Tissue-specific reductions in mitochondrial efficiency and increased ROS release rates during ageing in zebra finches, Taeniopygia guttata
Source: GeroScience. 2022 Aug 19;45(1):265–76. doi: 10.1007/s11357-022-00624-1 (PMC9886749; doi:10.1007/s11357-022-00624-1)
Supplement: Supplementary file 1 — Supplementary file1 (DOCX 424 KB) [file 11357_2022_624_MOESM1_ESM.docx]

#### Supplementary Information

Tissue-specific reduction in mitochondrial efficiency and increased ROS release rates from the liver and muscle of old zebra finches

*Pablo Salmón, Caroline Millet, Pat Monaghan, Colin Selman, and Neal J. Dawson*

**Results:**

**
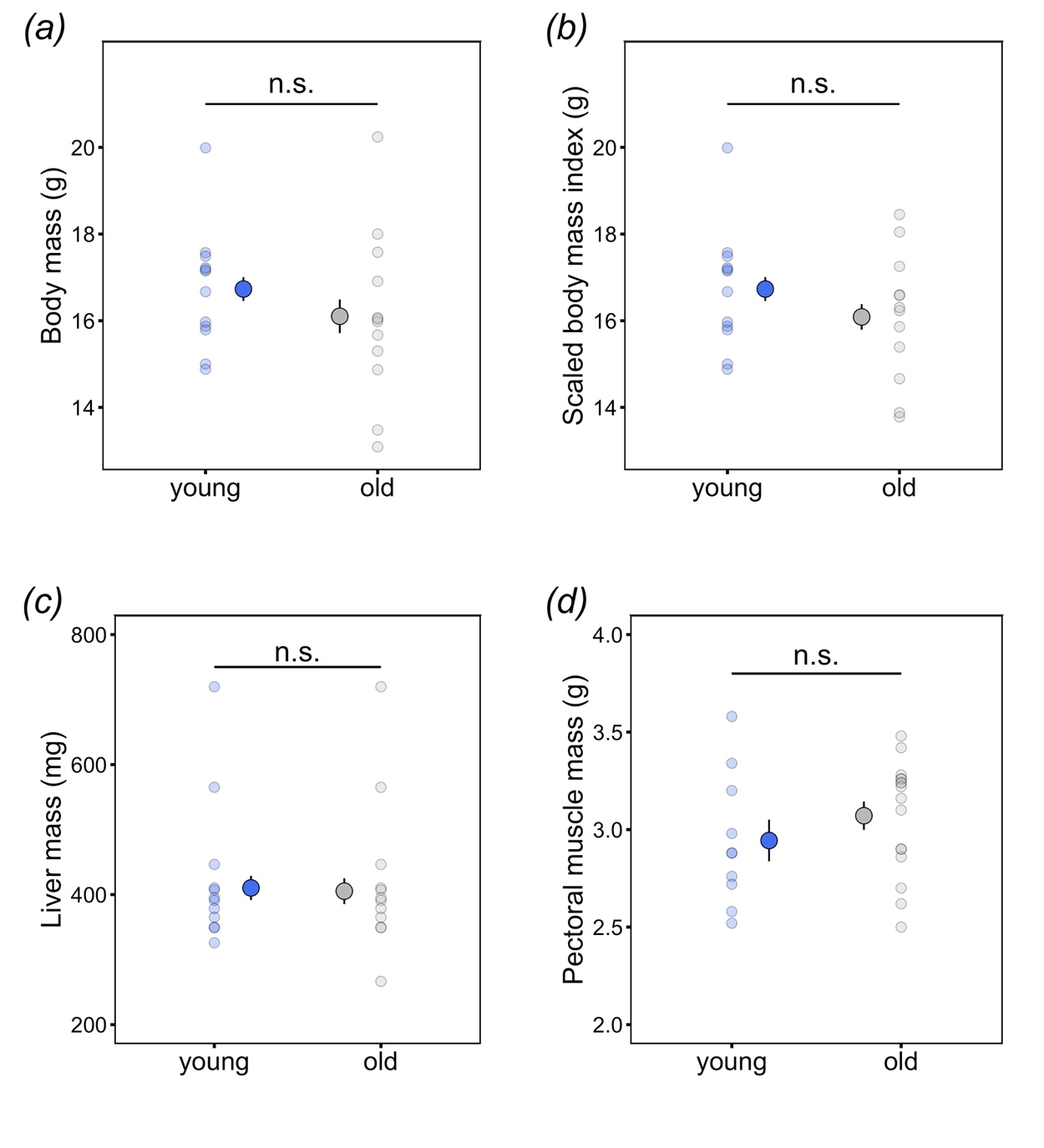
**

Figure S1. Phenotypic traits in 3 mo. and 4 yr old adult zebra finches for (a) body mass, (b) scaled body mass index, (c) total liver mass from young (blue) and old (grey) zebra finches. (d) Pectoral muscle (*pectoralis major*) mass was obtained from a seperate sample of zebra finches of similar ages (young, median = 78.4 days, range = 75-83 days; old, median = 1,392 days, range = 1,373-1,463 days). Light circles represent raw data points and dark circles represent means ± se per age group, n = 12 for young and old finches in panels *a-c*, n= 10 for young and n= 16 for old finches in panel *d* (see Supplementary table S1 for details).

**
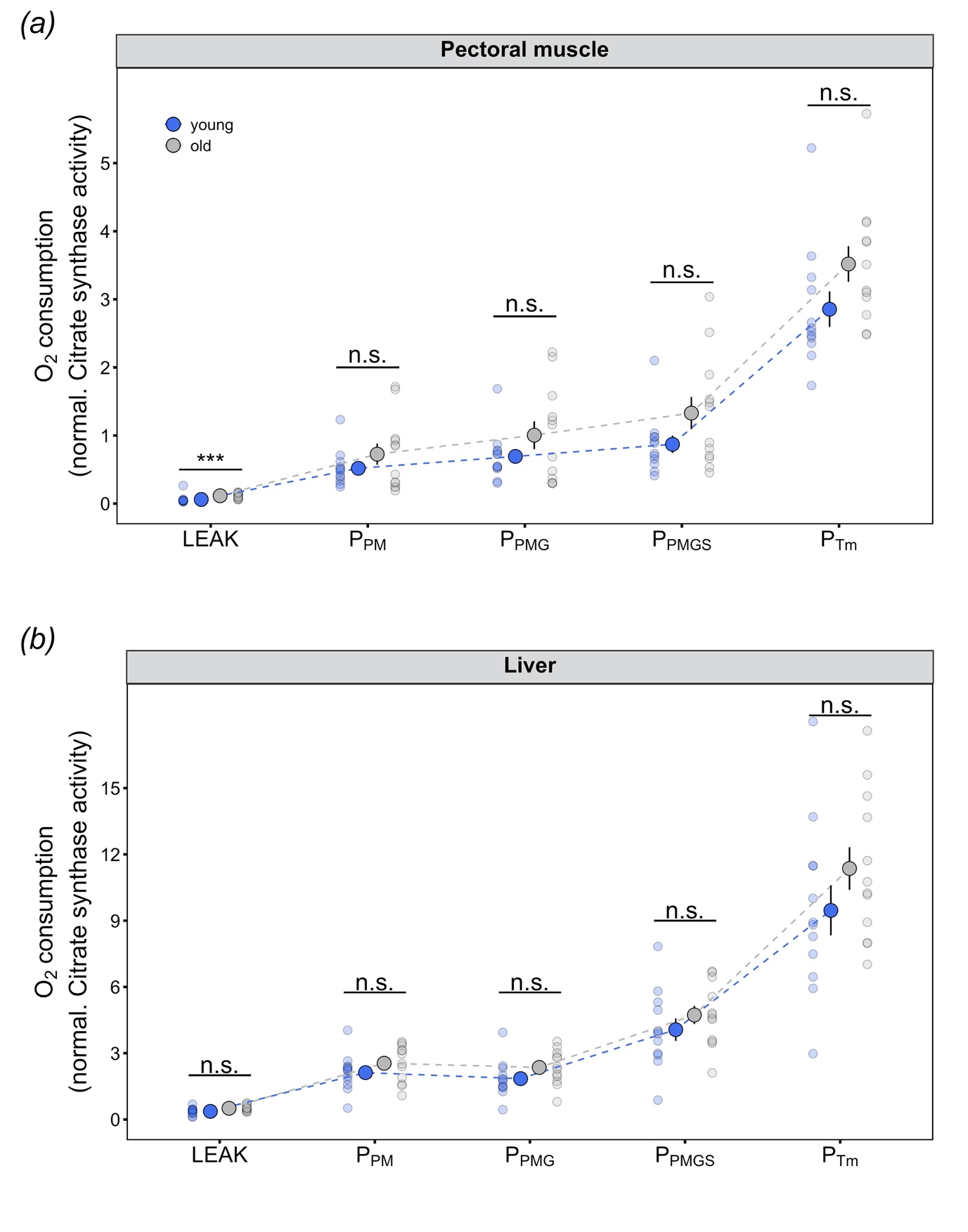
**Figure S2. Mitochondrial O2 consumption rates from young and old zebra finches normalised by citrate synthase activity (CS; mU/mg tissue) in (a) pectoral muscle and (b) liver tissues. LEAK = pyruvate + malate; PPM = pyruvate + malate + ADP; PPMG = pyruvate + malate + glutamate + ADP; PPMGS = pyruvate + malate + glutamate + succinate + ADP; PTm = TMPD + ascorbate. Blue circles = young finches; grey circles = old finches. Significant differences between age categories were assessed by Tukey-HSD test or main effects (see text and Supplementary table S2 for details), with *** P <*0.001*. Data are raw data points and means ± se, n=12 for both young and old finches.

Table S1. Summary of the linear mixed model for within-individual changes in mass and linear models for body mass, scaled mass index (SMI), liver mass in 3 mo.(young; n=12) and 4 yr (old; n=12) zebra finches. We also measured pectoral muscle (*pectoralis major*) mass in a seperate sample of zebra finches of similar ages (young, median = 78.4 days, range = 75-83 days, n=10; old, median = 1,392 days, range = 1,373-1,463 days, n=16). Est.= estimate, SE= standard error, df = degrees of freedom.

| **(a) Body mass** | Est. | SE | df | F | p-value |
| --- | --- | --- | --- | --- | --- |
| (Intercept) | 16.15 | 0.65 |  |  |  |
| Age category | 0.62 | 0.71 | 1, 21 | 0.79 | 0.384 |
| Sex | -0.07 | 0.71 | 1, 21 | 0.01 | 0.916 |
|  |  |  |  |  |  |
| **(b) Scaled Mass Index (SMI)** |  |  |  |  |  |
| (Intercept) | 16.23 | 0.55 |  |  |  |
| Age category | 0.62 | 0.60 | 1, 21 | 1.16 | 0.293 |
| Sex | -0.24 | 0.60 | 1, 21 | 0.17 | 0.683 |
|  |  |  |  |  |  |
| **(c) Liver mass** |  |  |  |  |  |
| (Intercept) | 416.99 | 43.94 |  |  |  |
| Age category | 5.42 | 47.96 | 1, 21 | 0.01 | 0.92 |
| Sex | 5.69 | 48.13 | 1, 21 | 0.01 | 0.91 |
|  |  |  |  |  |  |
| **(d) Pectoral muscle mass** |  |  |  |  |  |
| (Intercept) | 3.07 | 0.01 |  |  |  |
| Age category (young) | -0.13 | 0.09 | 1, 24 | 1.04 | 0.317 |

Table S2. Summary of the linear mixed models for mitochondrial O_2_ consumption rate (per mg tissue and normalised by Citrate synthase activity) in young and old zebra finches. O_2_ consumption values were log-transformed in both tissues in order to achieve model assumptions. Est.= estimate, SE= standard error, df = degrees of freedom, R^2^_m_= marginal R-squared (fixed effects only), R^2^_c_= conditional R-squared (whole model). P-values <0.05 are in bold and italics font.

|  | Muscle | | | | | Liver | | | | |
| --- | --- | --- | --- | --- | --- | --- | --- | --- | --- | --- |
| **O_2_ consumption** | Est. | SE | df | F | p-value | Est. | SE | df | F | p-value |
| (Intercept) | 1.81 | 0.19 |  |  |  | 2.52 | 0.13 |  |  |  |
| Age category (4yr) | 0.68 | 0.22 | 1,21 | 1.16 | 0.294 | 0.27 | 0.16 | 1,21 | 1.19 | 0.289 |
| Respiration state |  |  | 4,88 | 458.81 | ***<0.001*** |  |  | 4,88 | 692.55 | ***<0.001*** |
| P_PM_ | 2.29 | 0.13 |  |  |  | 1.77 | 0.09 |  |  | 73.740 |
| P_PMG_ | 2.58 | 0.13 |  |  |  | 1.62 | 0.09 |  |  | 100.470 |
| P_PMGS_ | 2.82 | 0.13 |  |  |  | 2.40 | 0.09 |  |  | 128.620 |
| P_Tm_ | 4.05 | 0.13 |  |  |  | 3.27 | 0.09 |  |  | 373.600 |
| Sex (Male) | -0.02 | 0.19 | 1,21 | 0.01 | 0.921 | 0.24 | 0.13 | 1,21 | 3.25 | 0.086 |
| Age category x respiration state |  |  | 4,88 | 4.51 | ***0.002*** |  |  | 4,88 | 0.73 | 0.571 |
| Age category (4 yr) x P_PM_ | -0.66 | 0.18 |  |  |  | -0.18 | 0.13 |  |  |  |
| Age category (4 yr) x P_PMG_ | -0.61 | 0.18 |  |  |  | -0.11 | 0.13 |  |  |  |
| Age category (4 yr) x P_PMGS_ | -0.48 | 0.18 |  |  |  | -0.18 | 0.13 |  |  |  |
| Age category (4 yr) x P_Tm_ | -0.60 | 0.18 |  |  |  | -0.16 | 0.13 |  |  |  |
|  |  | |  |  |  |  | |  |  |  |
| Model R^2^_m_ / R^2^_c_ | 0.83 /0.95 | |  |  |  | 0.89 / 0.96 | |  |  |  |
|  |  | |  |  |  |  | |  |  |  |
| **O_2_ consumption (CS normalised)** | Est. SE | | df | F | p-value | Est SE | | df | F | p-value |
| (Intercept) | -2.18 | 0.19 |  |  |  | -0.89 | 0.15 |  |  |  |
| Age category (4yr) | -0.82 | 0.22 | 1,21 | 3.57 | 0.0.73 | -0.37 | 0.17 | 1,21 | 2.64 | 0.119 |
| Respiration state |  |  | 4,88 | 458.81 | ***<0.001*** |  |  | 4,88 | 692.55 | ***<0.001*** |
| P_PM_ | 1.64 | 0.13 |  |  |  | 1.59 | 0.09 |  |  | 73.740 |
| P_PMG_ | 1.97 | 0.13 |  |  |  | 1.51 | 0.09 |  |  | 100.470 |
| P_PMGS_ | 2.34 | 0.13 |  |  |  | 2.22 | 0.09 |  |  | 128.620 |
| P_Tm_ | 3.46 | 0.13 |  |  |  | 3.11 | 0.09 |  |  | 373.600 |
| Sex (Male) | -0.07 | 0.19 | 1,21 | 0.13 | 0.720 | 0.30 | 0.15 | 1,21 | 4.15 | 0.054 |
| Age category x respiration state |  |  | 4,88 | 4.51 | ***0.002*** |  |  | 4,88 | 0.73 | 0.571 |
| Age category (4 yr) x P_PM_ | 0.66 | 0.18 |  |  |  | 0.18 | 0.13 |  |  |  |
| Age category (4 yr) x P_PMG_ | 0.61 | 0.18 |  |  |  | 0.11 | 0.13 |  |  |  |
| Age category (4 yr) x P_PMGS_ | 0.48 | 0.18 |  |  |  | 0.18 | 0.13 |  |  |  |
| Age category (4 yr) x P_Tm_ | 0.60 | 0.18 |  |  |  | 0.16 | 0.13 |  |  |  |
|  |  | |  |  |  |  | |  |  |  |
| Model R^2^_m_ / R^2^_c_ | 0.84 /0.95 | |  |  |  | 0.87 / 0.96 | |  |  |  |

Table S3. Summary of the linear mixed models for mitochondrial ROS release rate (H_2_O_2_) and ROS release/O_2_ consumption in young and old zebra finches. ROS release/O_2_ consumption was log-transformed in both tissues in order to achieve model assumptions. Est.= estimate, SE= standard error, df = degrees of freedom, R^2^_m_= marginal R-squared (fixed effects only), R^2^_c_= conditional R-squared (whole model). P-values <0.05 are in bold and italics font.

|  | Muscle | | | | | Liver | | | | |
| --- | --- | --- | --- | --- | --- | --- | --- | --- | --- | --- |
| **ROS release** | Est. | SE | df | F | p-value | Est. | SE | df | F | p-value |
| (Intercept) | 0.69 | 0.08 |  |  |  | 1.03 | 0.07 |  |  |  |
| Age category (4 yr) | -0.15 | 0.09 | 1,21 | 4.84 | ***0.039*** | -0.57 | 0.08 | 1,21 | 38.19 | ***<0.001*** |
| Respiration state |  |  | 3,66 | 47.83 | ***<0.001*** |  |  | 3,66 | 0.93 | 0.431 |
| P_PM_ | 0.03 | 0.03 |  |  |  | -0.05 | 0.02 |  |  | 73.740 |
| P_PMG_ | 0.19 | 0.03 |  |  |  | -0.07 | 0.02 |  |  | ###### |
| P_PMGS_ | 0.29 | 0.03 |  |  |  | -0.07 | 0.02 |  |  | ###### |
| Sex | -0.01 | 0.09 | 1,21 | 0.02 | 0.886 | 0.19 | 0.08 | 1,21 | 5.89 | ***0.024*** |
| Age category x respiration state |  |  | 3,66 | 3.28 | ***0.026*** |  |  | 3,66 | 10.44 | ***<0.001*** |
| Age category (4 yr) x P_PM_ | 0.01 | 0.04 |  |  |  | 0.08 | 0.03 |  |  |  |
| Age category (4 yr) x P_PMG_ | -0.09 | 0.04 |  |  |  | 0.10 | 0.03 |  |  |  |
| Age category (4 yr) x P_PMGS_ | -0.10 | 0.04 |  |  |  | 0.15 | 0.03 |  |  |  |
|  |  |  |  |  |  |  |  |  |  |  |
| Model R^2^_m_ / R^2^_c_ | 0.27 / 0.91 |  |  |  |  | 0.65 / 0.98 |  |  |  |  |
|  |  |  |  |  |  |  |  |  |  |  |
| **ROS release/O_2_ consumption** |  |  |  |  |  |  |  |  |  |  |
| (Intercept) | 1.70 | 0.20 |  |  |  | 1.79 | 0.14 |  |  |  |
| Age category (4 yr) | 0.42 | 0.23 | 1,21 | 0.02 | 0.908 | -0.44 | 0.16 | 1,21 | 9.38 | ***0.006*** |
| Respiration state |  |  | 3,66 | 268.47 | ***<0.001*** |  |  | 3,66 | 506.80 | ***<0.001*** |
| P_PM_ | -1.60 | 0.13 |  |  |  | -1.64 | 0.09 |  |  |  |
| P_PMG_ | -1.73 | 0.13 |  |  |  | -1.57 | 0.09 |  |  |  |
| P_PMGS_ | -2.00 | 0.13 |  |  |  | -2.29 | 0.09 |  |  |  |
| Sex | -0.01 | 0.21 | 1,21 | 0.001 | 0.97 | -0.04 | 0.15 | 1,21 | 0.06 | 0.810 |
| Age category x respiration state |  |  | 3,66 | 5.59 | ***0.002*** |  |  | 3,66 | 0.33 | 0.803 |
| Age category (4 yr) x P_PM_ | -0.60 | 0.18 |  |  |  | -0.07 | 0.12 |  |  |  |
| Age category (4 yr) x P_PMG_ | -0.66 | 0.18 |  |  |  | 0.03 | 0.12 |  |  |  |
| Age category (4 yr) x P_PMGS_ | -0.50 | 0.18 |  |  |  | 0.04 | 0.12 |  |  |  |
|  |  |  |  |  |  |  |  |  |  |  |
| Model R^2^_m_ / R^2^_c_ | 0.72 / 0.92 |  |  |  |  | 0.82 / 0.95 |  |  |  |  |
